# Safety and clinical activity of PD-L1 blockade in patients with aggressive recurrent respiratory papillomatosis

**DOI:** 10.1186/s40425-019-0603-3

**Published:** 2019-05-03

**Authors:** Clint T. Allen, Sunmin Lee, Scott M. Norberg, Damian Kovalovsky, Hong Ye, Paul E. Clavijo, Siwen Hu-Lieskovan, Richard Schlegel, Jeffrey Schlom, Julius Strauss, James L. Gulley, Jane Trepel, Christian S. Hinrichs

**Affiliations:** 1 Translational Tumor Immunology Program, National Institute of Deafness and Other Communication Disorders, National Institutes of Health, 10 Center Drive, Room 7N240C, Bethesda, MD 20892 USA; 2 Developmental Therapeutics Branch, National Cancer Institute, National Institutes of Health, Bethesda, USA; 3 Experimental Transplantation and Immunology Branch, National Cancer Institute, National Institutes of Health, Bethesda, USA; 4 University of California Los Angeles School of Medicine, Los Angeles, USA; 5 Georgetown University Medical Center, Washington, USA; 6 Laboratory of Tumor Immunology and Biology, National Cancer Institute, National Institutes of Health, Bethesda, USA; 7 Genitourinary Malignancies Branch, National Cancer Institute, National Institutes of Health, Bethesda, USA

**Keywords:** Immune checkpoint inhibition, Avelumab, Human papillomavirus, Recurrent respiratory papillomatosis

## Abstract

**Background:**

Recurrent respiratory papillomatosis (RRP) is a human papillomavirus (HPV)-driven disorder that causes substantial morbidity and can lead to fatal distal airway obstruction and post-obstructive pneumonias. Patients require frequent surgical debridement of disease, and no approved systemic adjuvant therapies exist.

**Methods:**

A phase II study was conducted to investigate the clinical activity and safety of programmed death-ligand 1 (PD-L1) blockade with avelumab in patients with RRP.

**Results:**

Twelve patients were treated. All patients with laryngeal RRP displayed improvement in disease burden, and 5 of 9 (56%) displayed partial responses. None of 4 patients with pulmonary RRP displayed a response. Using each patient’s surgical history as their own control, patients required fewer surgical interventions after avelumab treatment (*p* = 0.008). A subset of partial responders developed HPV-specific reactivity in papilloma-infiltrating T-cells that correlated with reduced HPV viral load and an increased Tissue Inflammation Signature.

**Conclusions:**

Avelumab demonstrated safety and clinical activity in patients with laryngeal RRP. Further study of immune checkpoint blockade for RRP, possibly with longer treatment duration or in combination with other immunotherapies aimed at activating antiviral immunity, is warranted.

**Trial registration:**

NCT, number NCT02859454, registered August 9, 2016.

**Electronic supplementary material:**

The online version of this article (10.1186/s40425-019-0603-3) contains supplementary material, which is available to authorized users.

## Background

Recurrent respiratory papillomatosis (RRP) is a clinical disorder characterized by aerodigestive tract papillomas that cause severe voice disturbance, airway obstruction, and potentially fatal distal small-airway obstruction leading to post-obstructive pneumonias [1, 2]. The incidence of RRP is approximately 2 per 100,000 in adults [3]. It is caused by chronic infection with low-risk human papillomavirus (HPV) types 6 or 11 [2, 4]. Surgical debridement of papillomas is the standard of care treatment approach, and patients with aggressive RRP can undergo hundreds of lifetime surgeries to control their disease. Advancements in laser surgical technologies have enhanced the ability to precisely remove RRP lesions, but lesion recurrence and the morbidity associated with both the disease itself and repeat surgical interventions remain problematic [5]. To date, no systemic adjuvant therapies are widely utilized for this difficult-to-treat disorder [6]. The clinical efficacy of systemic type I interferon in the treatment of RRP was previously studied [7], but widespread use of this treatment has been limited given lack of durable RRP lesion control and a high rate of adverse events (AEs).

Immune checkpoint inhibition (ICI) of the programmed death (PD)-axis has clinical activity in certain HPV-associated malignancies [8–10]. Programmed death-ligand 1 (PD-L1) blockade in RRP has not been reported [11]. Treatment of RRP with PD-L1 blockade is an attractive strategy given the presence of HPV DNA in the lesions and the likelihood that this DNA harbors one or more T cell antigens. RRP biopsy specimens display expression of PD-L1 by both papillomas and infiltrating immune cells [12], suggesting that the PD-signaling axis may play a role in suppression of effective immune responses. Here, we report clinical and immune correlative findings from a phase II clinical trial for the treatment of patients with aggressive RRP with avelumab, an FDA-approved anti-PD-L1 monoclonal antibody (mAb) [13, 14].

## Methods

### Patients

Patients age 18 years or older with a pathologically confirmed diagnosis of squamous papilloma were eligible for the trial. For laryngeal disease, eligibility required an anatomic Derkay score [15] of 10 or greater, and two or more surgical interventions to control papillomas in the prior 12 months. For pulmonary disease, eligibility required measurable lung lesions by RECIST1.1. Inclusion criteria included adequate organ function as assessed by standard laboratory tests and Eastern Cooperative Oncology Group performance status of 0 or 1. Exclusion criteria included infection with HIV, hepatitis B, or hepatitis C; a history of solid organ transplant or autoimmune disease; immunosuppression; and prior treatment with an immune checkpoint inhibitor.

### Study design

This study was designed as a phase II clinical trial. The protocol was approved by the National Cancer Institute (NCI) Institution Review Board and the National Institutes of Health Clinical Center, and informed consent was obtained from all patients. Treatment consisted of avelumab (10 mg/kg) iv every 2 weeks for three doses (one course). Patients who demonstrated a partial response (PR) after the first course were treated with a second course. Hence, patients could receive a total of up to six doses of avelumab over 12 weeks. Patients who did not demonstrate a PR after one course did not receive a second course. Following completion of treatment, all patients underwent surgical debridement of papillomas with standard-of-care techniques, and they returned to their referring otolaryngologist to resume standard-of-care treatment for their RRP. Avelumab was provided by EMD Serono through a Cooperative Research and Development Agreement with the NCI.

### Assessments

Clinical assessments including office-based endoscopy via flexible laryngoscopy and completion of the Voice Handicap Index-10 (VHI-10) questionnaire [16] were performed at the time of screening and prior to each dose of the study drug. Chest computed tomography (CT) evaluation was obtained on patients with known or suspected lung disease. Complete response was defined as complete resolution of all laryngeal papillomas visible on office-based endoscopy and all pulmonary papillomas evident by CT scan. PR for laryngeal disease was defined as a decrease in anatomic Derkay score of ≥50%. After four patients were treated, the definition was changed to ≥30% to lower the threshold for a second course of treatment as the treatment was well tolerated and appeared to have activity. Responses are reported based on the protocol criteria at the time of treatment. For pulmonary disease, response was defined using RECIST 1.1 criteria. All patients were examined under anesthesia prior to the first and second doses of avelumab to verify patency of the airway and to obtain biopsies. During the baseline and 2-week exams under anesthesia, < 1 mm biopsies were obtained under direct visualization from locations that would not change the Derkay score, such as tracheal or large supraglottic lesions. Normal mucosa was sampled from the posterior arytenoid or post-cricoid regions in all patients at all time points. Clinical laboratory tests were obtained weekly on patients throughout the duration of treatment. Determination of response was based upon changes in anatomic Derkay score obtained via office-based endoscopy and/or chest CT (when indicated) at 6 or 12 weeks.

### Outcomes

The primary outcome measure was rate of complete response. Secondary outcome measures included the rate of PR, change in inter-surgery interval, change in VHI-10, rate of adverse events, and change in immune correlatives.

### Immune correlative assays

T cell cultures were initiated from papillomas and adjacent but normal appearing laryngeal mucosa fragments using interleukin-2 containing media [17]. Expanded T-cells from papilloma and normal mucosa were tested for reactivity against autologous feeder cells (dendritic cells or B cells) transfected with mRNA from individual HPV genes. T-cell reactivity against these products or PMA/Ionomycin (positive control) was assessed via a standard ELISPOT assay. HPV 6 or 11 copy number per cell was determined using real-time quantitative PCR primers specific for HPV L1 (type specific) and Ribonuclease P. For immunohistochemistry, sectioned FFPE tissue samples were stained with haematoxylin and eosin, anti-CD8, and anti-PD-L1 at the UCLA Anatomic Pathology Immunohistochemistry and Histology Laboratory. Antibodies used included CD8 clone C8/144B (Dako, 1:100, low pH retrieval) and PD-L1 (Spring Biosciences, Sp142, 1:200, high pH retrieval). IHC was performed on a Leica Bond III autostainer. Percentage cellularity (% positive cells/all nucleated cells) was calculated using the Halo platform (Indica Labs). For gene expression analysis, RNA was extracted from frozen tissues and NanoString PanCancer IO 360 Gene Expression Panel™ analysis (NanoString Technologies, Seattle, WA) was performed per the manufacturer’s recommendations. Expression of individual HPV genes was determined by Droplet Digital™ PCR (ddPCR™) analysis (Bio-Rad, Hercules, CA) using HPV type and gene specific primers. Bioinformatic analysis was performed by NanoString.

### Statistical analysis

The Wilcoxon matched-pairs test was used to test for a significant difference in the surgery-free interval. A *p* value of < 0.05 was considered statistically significant.

## Results

### Patient characteristics

Twelve patients were treated (Table 1). The median age was 51 years (range 21 to 67 years). Four patients had juvenile onset RRP, and eight had adult onset RRP. The median number of years since diagnosis of RRP was 18 (range 2 to 45 years). Eligibility was based on laryngeal disease for eight patients, pulmonary disease for three patients, and both laryngeal and pulmonary disease for one patient. The median Derkay score of patients who qualified for the study based on laryngeal disease was 13 (range 10 to 26). Ten patients had required at least 20 surgeries to control their RRP lesions since initial diagnosis, and three patients had required greater than 100 surgeries. Most patients had received adjuvant local or systemic treatment with agents such as cidofovir or bevacizumab prior to enrollment. RRP was associated with HPV 11 in six patients and HPV 6 in six patients. There was a trend toward greater likelihood of experiencing a partial response to avelumab in patients with RRP associated with HPV 6 compared to HPV 11, although this failed to reach statistical significance in this small cohort (Additional file 1: Figure S1). There was no correlation between HPV subtype and the presence of pulmonary disease (Additional file 1: Figure S2).

**Table 1:** Characterization of Patient and Prior Treatments

Patient	Age (Years)	Sex	HPV type	Age at diagnosis (Years)	Number of RRP surgeries lifetime	Prior local treatments	Prior systemic treatments	Pulmonary RRP lesions
1	32	F	11	19	> 40	Cidofovir	I-3-C	No
2	36	M	6	28	35	Cidovofir, bevacizumab	I-3-C	No
3	50	F	11	5	> 250	Mitomycin	Diindolylmethane	Yes
4	63	M	11	19	> 50	Cidofovir, Cryotherapy	None	No
5	27	F	6	6	> 50	None	None	No
6	53	F	11	38	> 20	Cidofovir	None	Yes
7	55	M	6	53	11	Cidovofir, bevacizumab	None	No
8	55	F	6	51	16	None	None	No
9	56	M	11	54	> 20	Cidofovir	None	No
10	21	M	6	1	> 100	Cidovofir, bevacizumab	Interferon, Methotrexate	Yes
11	67	M	6	28	> 40	bevacizumab, PDT	I-3-C	Yes
12	26	M	11	2	> 100	Cidofovir, bevacizumab	Interferon, I-3-C	No

Abbreviations: *HPV* human papillomavirus, *RRP* recurrent respiratory papillomatosis, *PDT* photodynamic therapy, *I-3-C* indole-3-carbinol

### Clinical activity

**Fig. 1:**
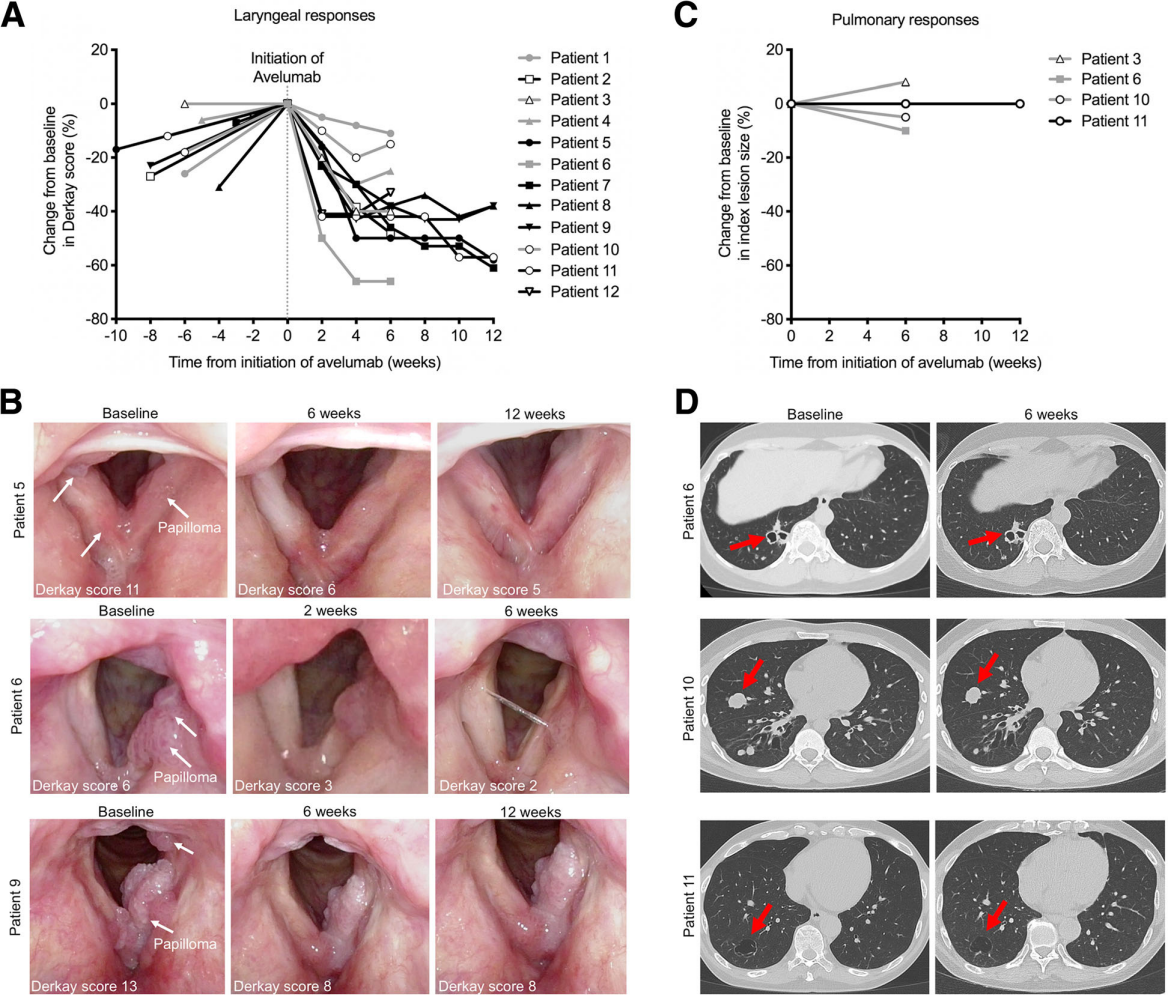
Clinical response following initiation of avelumab in patients with recurrent respiratory papillomatosis. **a**, A spider plot of change in laryngeal disease burden for each patient, as measured by anatomic Derkay score, is shown. The dotted line shows the time that avelumab was initiated. The negative time points reflect screening assessments prior to starting treatment. Patients 5, 7, 8, 9 and 11 received 6 doses of avelumab over 12 weeks. Patients 1, 2, 3, 4, 6, 10 and 12 received three doses over 6 weeks. **b**, Representative endoscopic images of RRP lesions for patients 5, 6 and 9 at the timepoints indicated are displayed. **c**, A spider plot of the change in pulmonary disease burden for each patient, as measured by CT scan imaging per RECIST1.1 guidelines, is shown. **d**, Representative images from CT scans from patients 6, 10 and 11 are displayed. Red arrows point to index lesions

Treatment with avelumab was associated with a decrease in Derkay score in all patients (Fig. 1a&b). Six of nine patients with qualifying (i.e. Derkay score ≥ 10) laryngeal disease experienced a partial response. Patient 2 received only one dose of avelumab due to laboratory abnormalities but demonstrated a 46% reduction in Derkay score. Another patient demonstrated a 33% reduction in Derkay score but received systemic steroids after completion of course one for an issue unrelated to the protocol, making him ineligible for further treatment. Pulmonary disease did not respond to treatment in any of the four patients with pulmonary disease (Fig. 1c&d). One patient with both laryngeal and pulmonary disease demonstrated a PR in the larynx but no response in the lung. No patients achieved a complete response. Three of twelve patients had received polyvalent HPV vaccine after their RRP diagnosis but prior to enrollment in this protocol. There was no correlation between patients who received the HPV vaccine and patients who experienced a partial response to avelumab (Additional file 1: Figure S3).

**Fig. 2:**
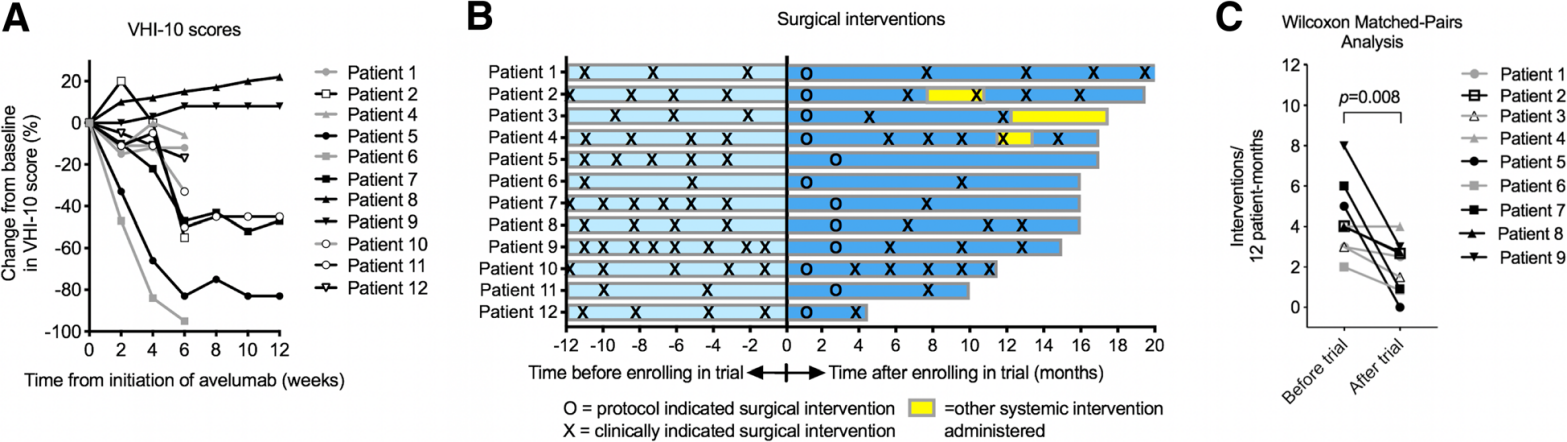
Changes in vocal function and surgery-free interval in patients with recurrent respiratory papillomatosis. **a**, A spider plot of change in VHI-10 scores is shown. **b**, A swimmer’s plot that demonstrates the frequency of surgical interventions before and after treatment with avelumab is shown. Each patient received a protocol indicated surgical intervention that occurred 6 or 12 weeks after initiation of avelumab at time zero. Yellow regions indicate the administration of a systemic therapy (anti-PD-1 mAb for patient 2, systemic bevacizumab for patients 3 and 4) for some patients after completing the protocol. **c**, The change in the number of clinically indicated surgical interventions per 12 patient-months before and after completion of the study. *P* = 0.008 by Wilcoxon matched-pairs analysis

The degree of papilloma present on the true vocal folds would be expected to determine vocal handicap. For patients with disease located on the true vocal folds, 9 of 11 patients reported decreased VHI-10 scores, indicating improved vocal function, while receiving avelumab treatment (Fig. 2a). Two patients who experienced worsening vocal function while on treatment demonstrated reduction in the overall Derkay score but not in true vocal fold disease burden with treatment. The number of clinically indicated surgical interventions after avelumab treatment was compared to each patient’s historical rate of interventions before treatment to assess for changes in surgery-free interval (Fig. 2b). This allowed each patient to serve as their own control and provides an individual measure of duration of response to avelumab. Among patients who had 12 months or more of follow-up data after completion of the trial at the time of analysis (*n* = 9, median 13.5 months), there were significantly fewer interventions per 12 patient-months required after avelumab treatment compared to before treatment (Fig. 2c, *p* = 0.008, Wilcoxon matched-pairs analysis). In the post-protocol follow-up period, one patient (patient 2) received a course of treatment with different PD-axis immune checkpoint inhibitor, and two patients (patients 3 and 4) received systemic bevacizumab. These data suggest that some patients experienced clinical benefit with improved vocal function and the need for fewer surgical interventions after avelumab treatment.

### Adverse events

There were no grade 3 or 4 AEs (Table 2). The most common AE was grade 1 or grade 2 dry mouth, which developed in 5 of 12 patients. All patients reported ≥50% subjective recovery of dry mouth within 9 months of cessation of avelumab treatment, without systemic steroid administration. One patient experienced a flare of facial eczema/dry skin (pre-existing diagnosis) following her second dose of avelumab that was controlled with topical steroids and lasted for approximately 3 months after completion of treatment. One patient experienced a grade 2 infusion reaction that interrupted the first infusion. He was able to complete the infusion and did not experience infusion reactions with subsequent avelumab treatments. Patient 2 demonstrated asymptomatic grade 2 leukopenia and neutropenia on clinical laboratory assessment after one dose of avelumab, and the decision was made to discontinue treatment. Leukocyte and neutrophil counts normalized within weeks without the use of systemic steroids.

**Table 2:** Summary of Adverse Events

	Grade			
	No. of patients (%)			
	1	2	3	4
Fatigue	4 (33)	0	0	0
Malaise	1 (8)	0	0	0
Fever	2 (17)	0	0	0
Chills	1 (8)	0	0	0
Tachycardia	1 (8)	0	0	0
Headache	3 (25)	0	0	0
Anemia	2 (17)	0	0	0
Leukopenia	1 (8)	1 (8)	0	0
Neutropenia	0	1 (8)	0	0
Nausea	2 (17)	0	0	0
Constipation	1 (8)	0	0	0
Dysgeusia	1 (8)	0	0	0
Hyperthyroid	2 (17)	0	0	0
Hyperbilirubinemia	1 (8)	0	0	0
Increase in amlyase	1 (8)	0	0	0
Increase in lipase	2 (17)	0	0	0
Dry skin	0	1 (8)	0	0
Infusion reaction	0	1 (8)	0	0
Dry mouth	3 (25)	2 (17)	0	0
Maculopapular rash	1 (8)	0	0	0

### Immune correlates

**Fig. 3:**
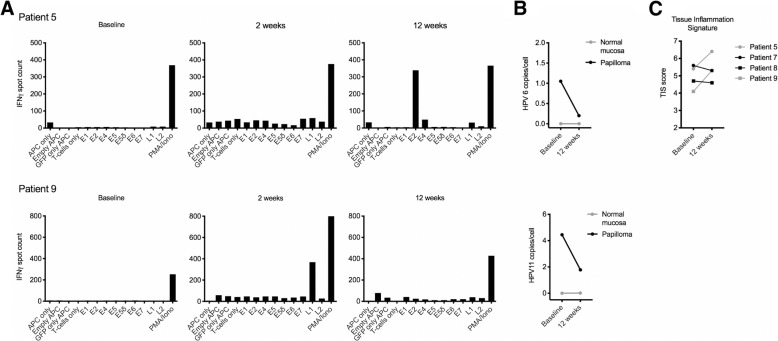
HPV-specific T-cell responses and viral burden in patients treated with avelumab. **a**, HPV-specific responses in papilloma-infiltrating T-cells from patients 5 and 9 were measured by interferon gamma enzyme-linked immunospot assays. Antigen presenting cells were loaded with RNA encoding individual transcripts of HPV 6 (patient 5) or HPV 11 (Patient 9) genes. Patients 7 and 8 did not demonstrate HPV-specific responses in papilloma-infiltrating T-cells. **b**, HPV viral load for HPV 6 (patient 5) or HPV 11 (Patient 9) in papilloma and normal mucosa was determined by quantitative-polymerase chain reaction. **c**, The Tissue Inflammation Signature was calculated from NanoString IO 360 analysis for patients 5, 7, 8, and 9

To study if PD-L1 immune checkpoint blockade induced anti-viral T cell responses in patients with clinical responses, four patients (patients 5, 7, 8 and 9) were selected (based on PR and tissue availability) for immune correlative analysis of tissues. Patient 5 demonstrated HPV6 E2-specific responses at the 12-week time point in T-cell cultures from papilloma but not normal mucosa (Fig. 3a). This correlated with a reduction in papilloma HPV6 viral load. Patient 9 demonstrated HPV11 L1-specific responses at the 2-week time point in papilloma but not normal mucosa, but this response was not detected at 12 weeks. This also correlated with a reduction in HPV11 viral load. Patients 7 and 8 did not demonstrate HPV-specific T-cell responses or a reduction in viral load in papilloma tissues. HPV gene expression within papillomas revealed consistently high expression of early genes E2, E4 and E5γ, with inconsistent E6 and E7 expression and low expression of late genes L1 and L2 (Additional file 1: Figure S4). HPV gene expression was not detected in the normal mucosa of any sample (Fig. 3b).

Immunohistochemistry was used to determine percentage of cells positive for CD8 or PD-L1 in papillomas from eleven of twelve patients with evaluable samples. There was a trend toward greater likelihood of experiencing a partial response in patients with higher percentage of CD8 and PD-L1 positive cells at baseline (Additional file 1: Figure S5). This failed to reach statistical significance in this small cohort. NanoString IO 360 analysis was used to investigate papilloma and normal samples for gene expression profiles related to tumor microenvironment, innate immune signaling and adaptive immunity (Additional file 1: Figure S6). Unsupervised clustering demonstrated segregation of papilloma from normal mucosa, with enrichment of papilloma gene expression indicative of increased proliferation, glycolytic metabolism and hypoxia. The tissue inflammation signature (TIS) [18], composed of an 18-gene expression panel validated for assessment of effector immune and interferon responses and predictive of a response to PD-based immune checkpoint blockade, was calculated from the NanoString IO 360 analysis. While the baseline TIS score did not predict response to avelumab, TIS was elevated in the post-treatment papilloma biopsies compared to baseline in the two patients who demonstrated HPV-specific T-cell responses (Fig. 3c). Although clear patterns of avelumab treatment-related changes within the papilloma samples were absent, papilloma samples generally displayed gene expression indicative of low dendritic cell and CD8 T-cell infiltration but increased myeloid cell inflammation and transforming growth factor-β (TGFβ) expression, compared to normal mucosa. Cumulatively, these data suggest that treatment with avelumab may induce HPV6- or HPV11-specific T-cell responses in a subset of patients with RRP.

## Discussion

Patients with RRP can require dozens to hundreds of lifetime surgeries to maintain a serviceable voice and patent airway, and pulmonary spread can lead to life-threatening distal airway obstruction and post-obstructive pneumonia [2]. There is presently no generally accepted systemic therapy. In the present study, avelumab showed safety and clinical activity in patients with RRP. Patients also showed improved vocal function (as measured by VHI-10 scores) and increased surgery-free interval after avelumab treatment, suggesting that this treatment may be of clinical benefit to some patients. This work also suggests that ICI should be investigated as a potential treatment strategy for other disorders caused by chronic HPV infection that are recalcitrant to anti-viral therapy [19] such as chondylomata and genitourinary tract intraepithelial neoplasias.

Immunotherapy with systemic type I interferon administration for patients with RRP was previously reported [7]. This intensive treatment regimen combining systemic IFNα and surgical debulking produced an initial decrease in RRP lesion recurrence, but the effect was not durable as RRP lesion recurrence in the treatment group rebounded to levels similar to control within 6 months of completing treatment. Further, significant toxicity was observed in patients treated with IFNα, limiting widespread application of this treatment approach for patients with severe RRP.

Avelumab showed good tolerability with few grade 1/2 AEs and no grade 3/4 AEs. Reported rates of grade 3/4 AEs in patients with recurrent/metastatic cancer range from 2 to 13% [13, 14, 20]. Avelumab treatment was also limited to 6 weeks in non-responders and 12 weeks in responders, which is a relatively short duration of treatment compared to the treatment of patients with recurrent/metastatic cancer [21]. The median time to onset of AEs following initiation of ICI treatment for patients with recurrent malignancy ranges from 5 to 15 weeks [22], which is not significantly dissimilar to duration of treatment reported here. Several patients appeared to have ongoing regression of RRP disease at the time of avelumab discontinuation. Whether longer duration of treatment would increase the rate of grade 3/4 AEs or could eventually induce complete resolution of RRP lesions is unknown.

RRP is caused by HPV type 6 or 11 infection [23]. We report consistently high expression of HPV E2 and lower, less consistent expression of E6 and E7 within papillomas, suggesting that HPV remained episomal and did not integrate into the host genome [4, 24]. This is consistent with previous reports demonstrating a low integration rate for low-risk HPV subtypes [25, 26]. This gene expression profile is different than that observed with HPV 16 which commonly integrates, leading to very high expression of E6 and E7 due to loss of E2 [4, 27]. Here, we demonstrated induction of HPV 6 E2 or HPV 11 L1-specific T-cell responses in two partial responders following avelumab treatment that were absent at baseline. These same patients also had an increased TIS [18], indicative of more global immune activation within their RRP lesions, and decreased HPV viral load. We did not demonstrate HPV-specific T-cell responses or decreased HPV viral load in two other partial responders. Although HPV viral load present within papillomas over time after completion of avelumab treatment could provide valuable insight into the duration of response, biopsies beyond the post-treatment biopsy were not obtained. The possibility exists that avelumab induced non-HPV but papilloma-specific T-cell responses in these two patients [28]. While not possible here due to challenges with establishing T-cell cultures from papilloma biopsies, comparing HPV-specific T-cell responses in responders and non-responders could provide further insight. Whether development of HPV-specific T-cell responses in HPV-driven lesions are required for durable responses to ICI or other forms of immunotherapy requires further study.

Prior work demonstrated CD8+ T-cell infiltration and PD-L1 expression within the majority of archived RRP specimens, providing a rationale for PD-based ICI [12]. We demonstrated a trend toward greater likelihood of response to avelumab with higher baseline CD8+ cell infiltration and PD-L1 positivity. It is possible this would reach statistical significance with a larger cohort. Blockade of the PD signaling axis reverses adaptive immune resistance but may not reverse other mediators of local immunosuppression within neoplasms [29]. RRP lesions harbor an immunosuppressive cytokine profile [30, 31], dysfunctional interferon signaling and major histocompatibility class I expression [32] and regulatory T-cells [33], all of which could suppress local effector immunity independent of PD immune checkpoint signaling. The results of our study demonstrated low dendritic cell, macrophage and CD8+ T-cell gene expression within RRP lesions compared to normal mucosa, suggesting that complex defects in both innate and adaptive immunity may be limiting anti-HPV responses. A subset of RRP lesions demonstrated gene expression indicative of increased neutrophil and inflammatory myeloid cell infiltration and TGFβ expression. Given the immunosuppressive role of these features in solid malignancies [34–36], combination immunotherapy targeting immunosuppressive myeloid cells or TGFβ along with PD-based ICI could enhance responses in patients with RRP.

While all patients with laryngeal RRP showed some degree of disease regression with avelumab treatment, no patients with pulmonary RRP showed disease regression. One patient with both laryngeal and pulmonary RRP had regression of laryngeal RRP but not pulmonary RRP. Mechanisms of this possible differential response between laryngeal and pulmonary RRP are unclear, and it is difficult to acquire tissue from pulmonary lesions for study given their deep location and cystic nature. If this lack of pulmonary response to ICI is verified in future trials, gaining insight into why these lesions do not respond will be a critical focus of research since the majority of RRP-related mortality is associated with complications of pulmonary RRP [2].

Several study limitations exist. The drug was administered for a defined, short course, which decreased drug exposure and the risk of adverse events but may have limited efficacy. In addition, all patients with laryngeal disease demonstrated some level of disease improvement, which prevents the study of factors that contribute to disease response versus disease resistance. Finally, while clinical activity was demonstrated, the small sample size and short duration of follow-up limits conclusions about clinical benefit.

Future clinical development of ICI in RRP could include a longer treatment course, or combination treatment with an agent possessing a complementary mechanism of action such as TGFβ blockade or induction of type I interferon responses. Duration of treatment with ICI in patients who respond will be a balance between clinical benefit and risk of developing one or more clinically significant immune-related adverse events. A clearer understanding of whether RRP lesions harbor genomic alterations similar to malignancies that could render populations of cells resistant to T cell recognition may inform future immunotherapy strategies.

## Conclusions

In conclusion, this work establishes the safety profile and indicates a level of clinical activity for avelumab in patients with RRP. Future investigation of systemic treatment of RRP with PD-L1 blockade is warranted.

## Additional file

Additional file 1: **Figure S1.** Contingency plot of HPV subtype and development of a partial response to avelumab. Assessed for statistical significance with the Fisher’s exact test. **Figure S2.** Contingency plot of HPV subtype and the presence of pulmonary disease. Assessed for statistical significance with the Fisher’s exact test. **Figure S3.** Contingency plot of patients who received polyvalent HPV vaccine prior to enrollment and patients who experienced a partial response to avelumab. Assessed for statistical significance with the Fisher’s exact test. **Figure S4.** Expression of individual HPV 6 or 11 genes. Expression of individual HPV 6 (patients 5, 7, and 8) or HPV 11 (patient 9) genes in papilloma and normal mucosa was measured by droplet digital polymerase chain reaction. **Figure S5.** Baseline representative immunohistochemistry photomicrographs and quantification of percentage of papilloma infiltrating CD8+ cells and total PD-L1+ cells. Percentage of positive cells inset within photomicrographs. Quantification of staining in eleven of twelve patients with evaluable biopsies represent percentage of total cells in the entire section. Assessed for statistical significance with the Mann-Whitney test. **Figure S6.** Gene expression profiling of papillomas and normal tissue. A heatmap demonstrating unsupervised hierarchical clustering of expression of individual genes or gene signatures is shown. Gene expression was measured by NanoString IO 360 analysis in pre-treatment (pre), on-treatment (2wk) and post-treatment (off) papilloma and normal mucosa biopsies. Each horizontal row represents a separate biopsy. Each unit increase of expression on the heatmap scale is a doubling of the biologic process it represents. The red box highlights differences in TGFβ gene expression between papilloma and normal mucosa biopsies. (DOCX 3090 kb)

## Abbreviations

AE: Adverse event
CT: Computed tomography
ddPCR: Droplet Digital™ PCR
HPV: Human papillomavirus
ICI: Immune checkpoint inhibition
NCI: National Cancer Institute
PD: Programmed death
PD-L1: Programmed death-ligand 1
PR: Partial response
RRP: Recurrent respiratory papillomatosis
TGFβ: Transforming growth factor-β
VHI-10: Voice handicap index-10
