# Supplementary material for: Safety and clinical activity of PD-L1 blockade in patients with aggressive recurrent respiratory papillomatosis
Source: J Immunother Cancer. 2019 May 3;7:119. doi: 10.1186/s40425-019-0603-3 (PMC6500000; doi:10.1186/s40425-019-0603-3)
Supplement: Supplementary file 1 — Figure S1. Contingency plot of HPV subtype and development of a partial response to avelumab. Assessed for statistical significance with the Fisher’s exact test. Figure S2. Contingency plot of HPV subtype and the presence of pulmonary disease. Assessed for statistical significance with the Fisher’s exact test. Figure S3. Contingency plot of patients who received polyvalent HPV vaccine prior to enrollment and patients who experienced a partial response to avelumab. Assessed for statistical significance with the Fisher’s exact test. Figure S4. Expression of individual HPV 6 or 11 genes. Expression of individual HPV 6 (patients 5, 7, and 8) or HPV 11 (patient 9) genes in papilloma and normal mucosa was measured by droplet digital polymerase chain reaction. Figure S5. Baseline representative immunohistochemistry photomicrographs and quantification of percentage of papilloma infiltrating CD8+ cells and total PD-L1+ cells. Percentage of positive cells inset within photomicrographs. Quantification of staining in eleven of twelve patients with evaluable biopsies represent percentage of total cells in the entire section. Assessed for statistical significance with the Mann-Whitney test. Figure S6. Gene expression profiling of papillomas and normal tissue. A heatmap demonstrating unsupervised hierarchical clustering of expression of individual genes or gene signatures is shown. Gene expression was measured by NanoString IO 360 analysis in pre-treatment (pre), on-treatment (2wk) and post-treatment (off) papilloma and normal mucosa biopsies. Each horizontal row represents a separate biopsy. Each unit increase of expression on the heatmap scale is a doubling of the biologic process it represents. The red box highlights differences in TGFβ gene expression between papilloma and normal mucosa biopsies. (DOCX 3090 kb) [file 40425_2019_603_MOESM1_ESM.docx]

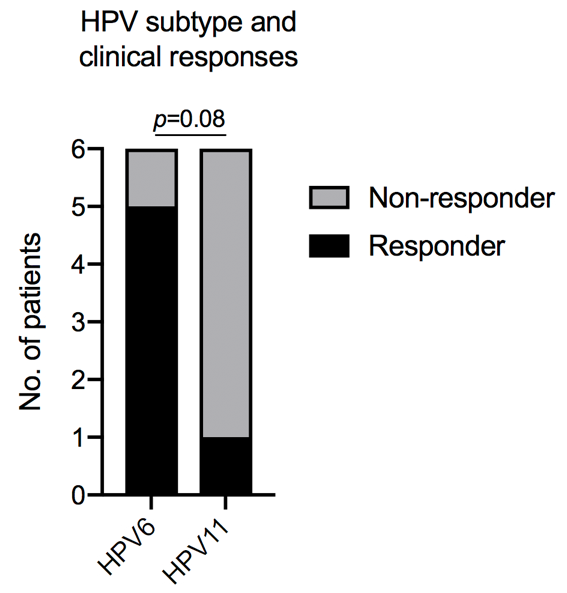


Supplemental Figure S1. Contingency plot of HPV subtype and development of a partial response to avelumab. Assessed for statistical significance with the Fisher’s exact test.


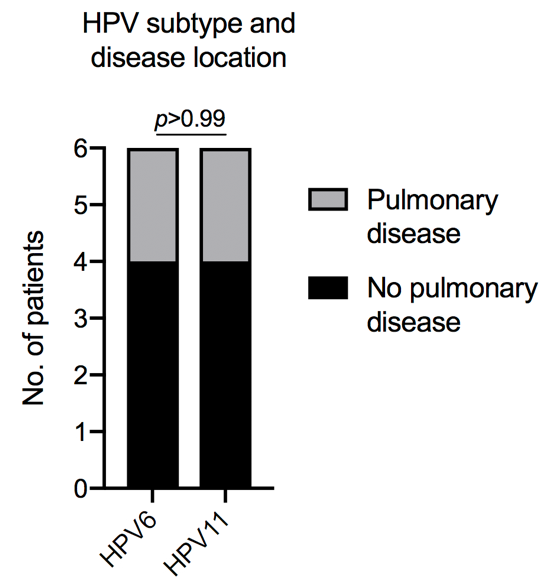


Supplemental Figure S2. Contingency plot of HPV subtype and the presence of pulmonary disease. Assessed for statistical significance with the Fisher’s exact test.


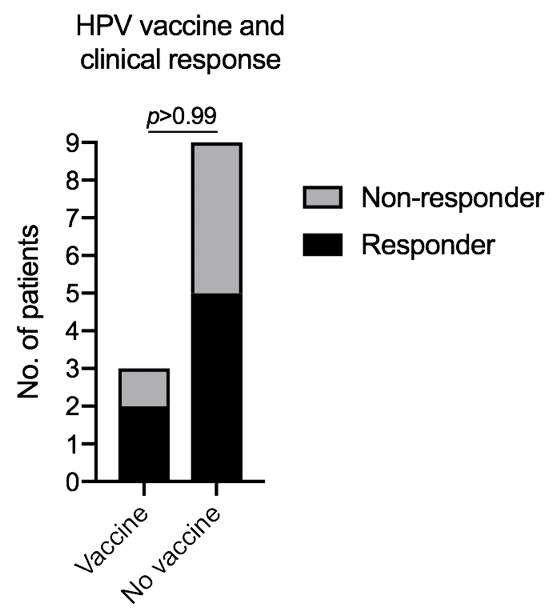


Supplemental Figure S3. Contingency plot of patients who received polyvalent HPV vaccine prior to enrollment and patients who experienced a partial response to avelumab. Assessed for statistical significance with the Fisher’s exact test.


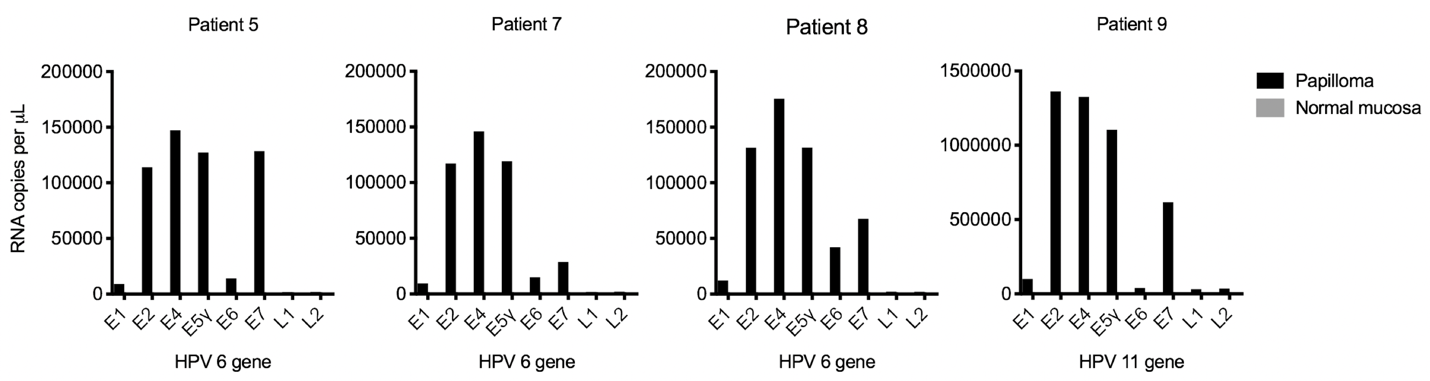


Supplemental Figure S4. Expression of individual HPV 6 or 11 genes. Expression of individual HPV 6 (patients 5, 7, and 8) or HPV 11 (patient 9) genes in papilloma and normal mucosa was measured by droplet digital polymerase chain reaction.


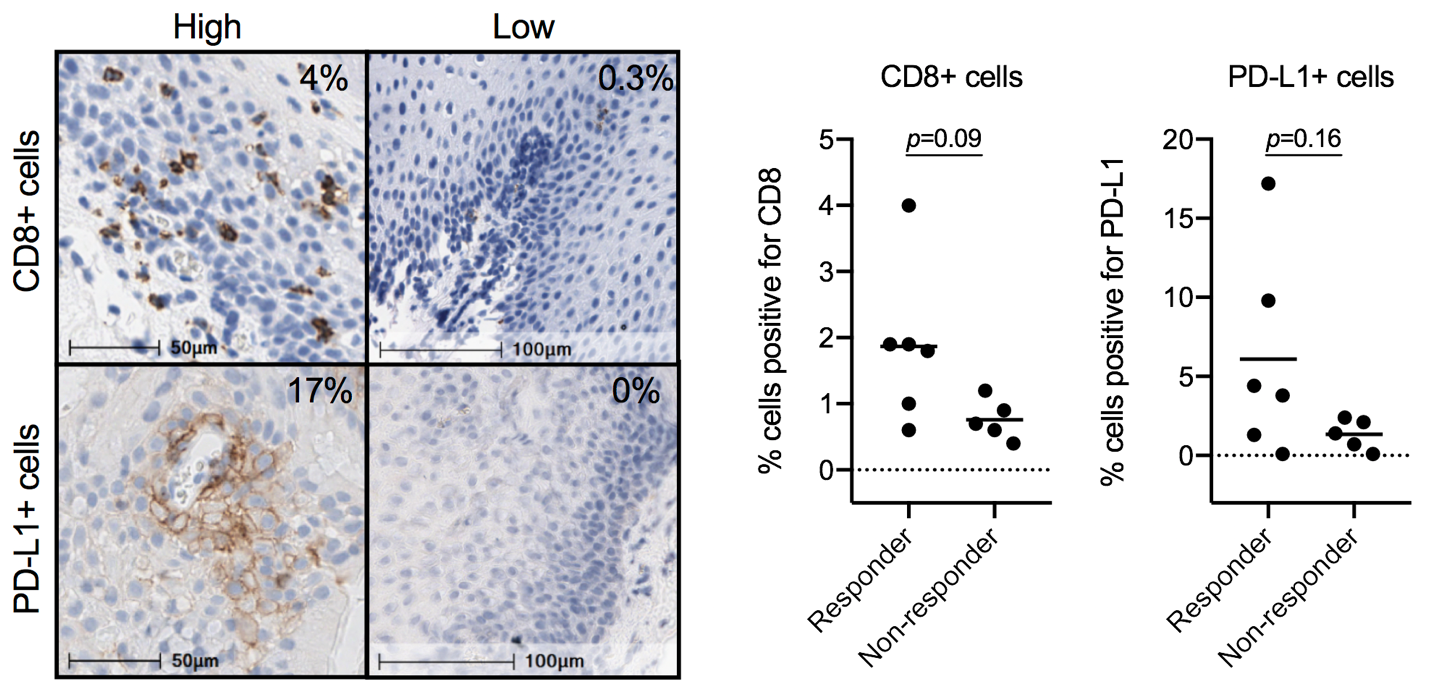


Supplemental Figure S5. Baseline representative immunohistochemistry photomicrographs and quantification of percentage of papilloma infiltrating CD8+ cells and total PD-L1+ cells. Percentage of positive cells inset within photomicrographs. Quantification of staining in eleven of twelve patients with evaluable biopsies represent percentage of total cells in the entire section. Assessed for statistical significance with the Mann-Whitney test.


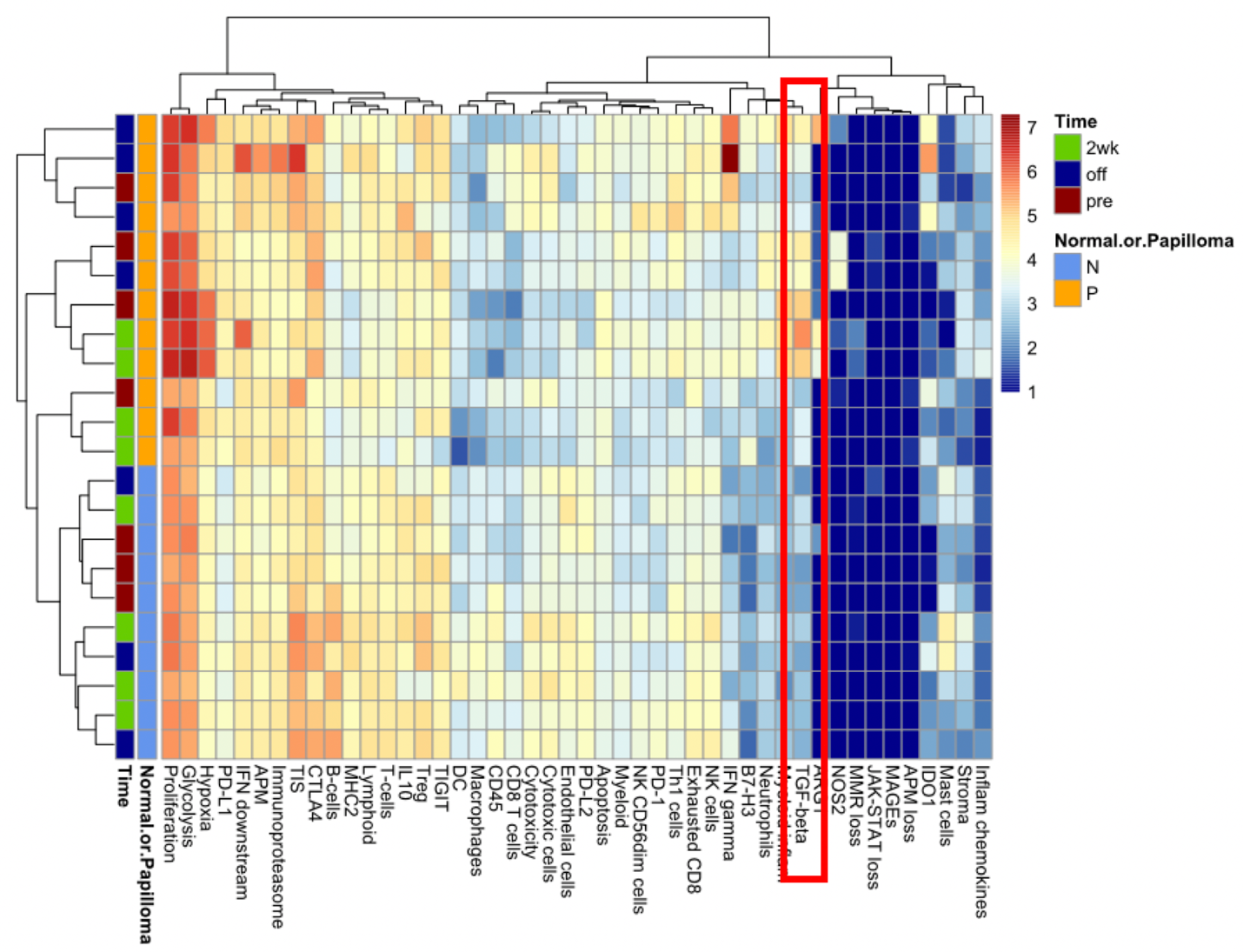


Supplemental Figure S6. Gene expression profiling of papillomas and normal tissue. A heatmap demonstrating unsupervised hierarchical clustering of expression of individual genes or gene signatures is shown. Gene expression was measured by NanoString IO 360 analysis in pre-treatment (pre), on-treatment (2wk) and post-treatment (off) papilloma and normal mucosa biopsies. Each horizontal row represents a separate biopsy. Each unit increase of expression on the heatmap scale is a doubling of the biologic process it represents. The red box highlights differences in TGFβ gene expression between papilloma and normal mucosa biopsies.
